# Current Awareness on Comparative and Functional Genomics

**DOI:** 10.1002/cfg.351

**Published:** 2004-03


**Comparative and Functional Genomics**
*Comp Funct Genom* 2004; **5**: 209–214
Published online in Wiley InterScience (www.interscience.wiley.com). **DOI:** 10.1002/cfg.351

### Current awareness on comparative and functional genomics

### 1 Reviews & symposia

Burke W. 2003. Genomics as a probe for disease biology (Review). *N Engl J Med* **349:** (10) 969.
Chakravarthy B, Pietenpol JA. 2003. Combined modality management of breast cancer: Development of predictive markers through proteomics. *Semin Oncol* **30:** (4 Suppl 9) 23.
Davisson RL. 2003. Physiological genomic analysis of the brain renin-angiotensin system (Review). *Am J Physiol* **285:** (3) R498.
Du C, Buckler E, Muse S. 2003. Development of a maize molecular evolutionary genomic database (Conference review). *Comp Funct Genom* **4:** (2) 246.
Dvornyk V, Recker RR, Deng HW. 2003. Gene expression studies of osteoporosis: Implications for microarray research (Review). *Osteoporosis Int* **14:** (6) 451.
Feder ME, Mitchell-Olds T. 2003. Evolutionary and ecological functional genomics. *Nat Rev Genet* **4:** (8) 651.
Galbraith DW. 2003. Global analysis of cell type-specific gene expression (Conference review). *Comp Funct Genom* **4:** (2) 208.
Giordano TJ. 2003. Gene expression profiling of endocrine tumors using DNA microarrays: Progress and promise (Review). *Endocr Pathol* **14:** (2) 107.
Gollery M. 2003. Specialized hidden Markov model databases for microbial genomics (Conference review). *Comp Funct Genom* **4:** (2) 250.
Gregory WF, Parkinson J. 2003. *Caenorhabditis elegans*: Applications to nematode genomics. *Comp Funct Genom* **4:** (2) 194.
Guerreiro N, Staedtler F, Grenet O, Kehren J, Chibout SD. 2003. Toxicogenomics in drug development. *Toxicol Pathol* **31:** (5) 471.
Hadjantonakis AK, Dickinson ME, Fraser SE, Papaioannou VE. 2003. Technicolour transgenics: Imaging tools for functional genomics in the mouse (Review). *Nat Rev Genet* **4:** (8) 613.
He QY, Chiu JF. 2003. Proteomics in biomarker discovery and drug development. *J Cell Biochem* **89:** (5) 868.
Horton WA. 2003. Skeletal development: Insights from targeting the mouse genome. *Lancet* **362:** (9383) 560.
Howbrook DN, Van der Valk AM, O'Shaughnessy MC, Sarker DK, Baker SC, Lloyd AW. 2003. Developments in microarray technologies (Review). *Drug Discov Today* **8:** (14) 642.
Levene AP, Morgan GJ, Davies FE. 2003. The use of genetic microarray analysis to classify and predict prognosis in haematological malignancies (Review). *Clin Lab Haematol* **25:** (4) 209.
Liu Z. 2003. A review of catfish genomics: Progress and perspectives (Conference review). *Comp Funct Genom* **4:** (2) 259.
Lloyd RV, Jin L, Kobayashi I. 2003. Advances in pituitary pathology: Pituitary tumor gene expression profiles (Review). *Endocrinologist* **12:** (4) 347.
Plomion C, Cooke J, Richardson T, Mackay J, Tuskan G. 2003. Report on the Forest Trees Workshop at the Plant and Animal Genome Conference (Conference review). *Comp Funct Genom* **4:** (2) 229.
Rolfe BG, Mathesius U, Djordjevic M, Weinman J, Hocart C, Weiller G, Dietz-Bauer W. 2003. Proteomic analysis of legume-microbe interactions (Conference review). *Comp Funct Genom* **4:** (2) 225.
Rom WN, Tchou-Wong KM. 2003. Functional genomics in lung cancer and biomarker detection. *Am J Respir Cell Molec Biol* **29:** (2) 153.
Rothschild MF. 2003. Advances in pig genomics and functional gene discovery (Conference review). *Comp Funct Genom* **4:** (2) 266.
Rudd S. 2003. Expressed sequence tags: Alternative or complement to whole genome sequences? *Trends Plant Sci* **8:** (7) 321.
Satoh N, Satou Y, Davidson B, Levine M. 2003. *Ciona intestinalis*: An emerging model for whole-genome analyses (Review). *Trends Genet* **19:** (7) 376.
Schoof H. 2003. Towards interoperability in genome databases: The MAtDB (MIPS *Arabidopsis thaliana* database) experience (Conference review). *Comp Funct Genom* **4:** (2) 255.
Searls DB. 2003. Pharmacophylogenomics: Genes, evolution and drug targets (Review). *Nat Rev Drug Discov* **2:** (8) 613.
Sebat JL, Colwell FS, Crawford RL. 2003. Metagenomic profiling: Microarray analysis of an environmental genomic library. *Appl Environ Microbiol* **69:** (8) 4927.
Terwilliger TC, Park MS, Waldo GS, Berendzen J, Hung LW, Kim CY, Smith CV, Sacchettini JC, Bellinzoni M, Bossi R *et al*. 2003. The TB structural genomics consortium: A resource for *Mycobacterium tuberculosis* biology (Review). *Tuberculosis* **83:** (4) 223.
van Steensel B, Henikoff S. 2003. Epigenomic profiling using microarrays (Review). *Biotechniques* **35:** (2) 346.
Wullschleger SD, Difazio SP. 2003. Emerging use of gene expression microarrays in plant physiology (Conference review). *Comp Funct Genom* **4:** (2) 216.
Wurtele ES, Li J, Diao L, Zhang H, Foster CM, Fatland B, Dickerson J, Brown A, Cox Z, Cook D, Lee EK, Hofmann H. 2003. MetNet: Software to build and model the biogenetic lattice of *Arabidopsis* (Conference review). *Comp Funct Genom* **4:** (2) 239.
Xue YB, Li JY, Xu ZH. 2003. Recent highlights of the China rice functional genomics program (Review). *Trends Genet* **19:** (7) 390.
Yeatman TJ. 2003. The future of cancer management: Translating the genome, transcriptome, and proteome (Review). *Ann Surg Oncol* **10:** (1) 7.

### 3 Large-scale sequencing and mapping

Clark M. 2003. Genomics and mapping of Teleostei (bony fish). *Comp Funct Genom* **4:** (2) 182.
Dufresne A, Salanoubat M, Partensky F, Artiguenave F, Axmann IM, Barbe V, Duprat S, Galperin MY, Koonin EV, Le Gall F *et al*. 2003. Genome sequence of the cyanobacterium *Prochlorococcus marinus* SS120, a nearly minimal oxyphototrophic genome. *Proc Natl Acad Sci U S A* **100:** (17) 10020.
Palenik B, Brahamsha B, Larimer FW, Land M, Hauser L, Chain P, Lamerdin J, Regala W, Allen EE, McCarren J *et al*. 2003. The genome of a motile marine *Synechococcus*. *Nature* **424:** (6952) 1037.

### 4 Evolutionary genomics

Garcia E, Elliott JM, Ramanculov E, Chain PSG, Chu MC, Molineux IJ. 2003. The genome sequence of *Yersinia pestis* bacteriophage $\phi$A1122 reveals an intimate history with the coliphage T3 and T7 genomes. *J Bacteriol* **185:** (17) 5248.
Murata Y, Nikaido M, Sasaki T, Cao Y, Fukumoto Y, Hasegawa M, Okada N. 2003. Afrotherian phylogeny as inferred from complete mitochondrial genomes. *Mol Phylogenet Evol* **28:** (2) 253.
Phillips MJ, Penny D. 2003. The root of the mammalian tree inferred from whole mitochondrial genomes. *Mol Phylogenet Evol* **28:** (2) 171.
Richly E, Chinnery PF, Leister D. 2003. Evolutionary diversification of mitochondrial proteomes: Implications for human disease. *Trends Genet* **19:** (7) 356.

In order to keep subscribers up-to-date with the latest developments in their field, this current awareness service is provided by John Wiley & Sons and contains newly-published material on comparative and functional genomics. Each bibliography is divided into 16 sections. 1 Reviews & symposia; 2 General; 3 Large-scale sequencing and mapping; 4 Evolutionary genomics; 5 Comparative genomics; 6 Pathways, gene families and regulons; 7 Pharmacogenomics; 8 EST, cDNA and other clone resources; 9 Functional genomics; 10 Transcriptomics; 11 Proteomics; 12 Protein structural genomics; 13 Metabolomics; 14 Genomic approaches to development; 15 Technological advances; 16 Bioinformatics. Within each section, articles are listed in alphabetical order with respect to author. If, in the preceding period, no publications are located relevant to any one of these headings, that section will be omitted.

Copyright © 2004 John Wiley & Sons, Ltd.

### 5 Comparative genomics

Anjum MF, Lucchini S, Thompson A, Hinton JCD, Woodward MJ. 2003. Comparative genomic indexing reveals the phylogenomics of *Escherichia coli* pathogens. *Infect Immun* **71:** (8) 4674.
Chambers EW, Lovin DD, Severson DW. 2003. Utility of comparative anchor-tagged sequences as physical anchors for comparative genome analysis among the Culicidae. *Am J Trop Med Hyg* **69:** (1) 98.
Gil R, Silva FJ, Zientz E, Delmotte F, Gonzalez-Candelas F, Latorre A, Rausell C, Kamerbeek J, Gadau J, Holldobler B *et al*. 2003. The genome sequence of *Blochmannia floridanus*: Comparative analysis of reduced genomes. *Proc Natl Acad Sci U S A* **100:** (16) 9388.
Hoopengardner B, Bhalla T, Staber C, Reenan R. 2003. Nervous system targets of RNA editing identified by comparative genomics. *Science* **301:** (5634) 832.
Hu M, Chilton NB, El-Osta YGA, Gasser RB. 2003. Comparative analysis of mitochondrial genome data for *Necator americanus* from two endemic regions reveals substantial genetic variation. *Int J Parasitol* **33:** (9) 955.
La D, Silver M, Edgar RC, Livesay DR. 2003. Using motif-based methods in multiple genome analyses: A case study comparing orthologous mesophilic and thermophilic proteins. *Biochemistry* **42:** (30) 8988.
Larkin DM, Everts-van der Wind A, Rebeiz M, Schweitzer PA, Bachman S, Green C, Wright CL, Campos EJ, Benson LD, Edwards J *et al*. 2003. A cattle-human comparative map built with cattle BAC-ends and human genome sequence. *Genome Res* **13:** (8) 1966.
Rocap G, Larimer FW, Lamerdin J, Malfatti S, Chain P, Ahlgren NA, Arellano A, Coleman M, Hauser L, Hess WR *et al*. 2003. Genome divergence in two *Prochlorococcus* ecotypes reflects oceanic niche differentiation. *Nature* **424:** (6952) 1042.
Sorrells ME, La Rota M, Bermudez-Kandianis CE, Greene RA, Kantety R, Munkvold JD, Miftahudin, Mahmoud A, Ma XF, Gustafson PJ *et al*. 2003. Comparative DNA sequence analysis of wheat and rice genomes. *Genome Res* **13:** (8) 1818.
Thomas JW, Touchman JW, Blakesley RW, Bouffard GG, Beckstrom-Sternberg SM, Margulies EH, Blanchette M, Siepel AC, Thomas PJ, McDowell JC *et al*. 2003. Comparative analyses of multi-species sequences from targeted genomic regions. *Nature* **424:** (6950) 788.

### 6 Pathways, gene families and regulons

Le Bras M, Benssad K, Soussi T. 2003. Data mining the p53 pathway in the *Fugu* genome: Evidence for strong conservation of the apoptotic pathway. *Oncogene* **22:** (32) 5082.
Li XN, Ding YQ, Liu GB. 2003. Transcriptional gene expression profiles of HGF/SF-met signaling pathway in colorectal carcinoma. *World J Gastroenterol* **9:** (8) 1734.
Panina EM, Mironov AA, Gelfand MS. 2003. Comparative genomics of bacterial zinc regulons: Enhanced ion transport, pathogenesis, and rearrangement of ribosomal proteins. *Proc Natl Acad Sci U S A* **100:** (17) 9912.

### 7 Pharmacogenomics

Adjei AA, Thomae BA, Prondzinski JL, Eckloff BW, Wieben ED, Weinshilboum RM. 2003. Human estrogen sulfotransferase (SULT1E1) pharmacogenomics: Gene resequencing and functional genomics. *Br J Pharmacol* **139:** (8) 1373.
Antonucci F, Chilosi M, Parolini C, Hamdan M, Astner H, Righetti PG. 2003. Two-dimensional molecular profiling of mantle cell 2 lymphoma. *Electrophoresis* **24:** (14) 2376.
Ariel N, Zvi A, Makarova KS, Chitlaru T, Elhanany E, Velan B, Cohen S, Friedlander AM, Shafferman A. 2003. Genome-based bioinformatic selection of chromosomal *Bacillus anthracis* putative vaccine candidates coupled with proteomic identification of surface-associated antigens. *Infect Immun* **71:** (8) 4563.
Beauchamp NJ, Van Achterbert TAE, Engelse MA, Pannekoek H, De Vries CJM. 2003. Gene expression profiling of resting and activated vascular smooth muscle cells by serial analysis of gene expression and clustering analysis. *Genomics* **82:** (3) 288.
Beer DG, Kardia SLR, Huang CC, Giordano TJ, Levin AM, Misek DE, Lin L, Chen GA, Gharib TG, Thomas DG *et al*. 2002. Gene-expression profiles predict survival of patients with lung adenocarcinoma. *Nat Med* **8:** (8) 816.
Catherino WH, Prupas C, Tsibris JCM, Leppert PC, Payson M, Nieman LK, Segars JH. 2003. Strategy for elucidating differentially expressed genes in leiomyomata identified by microarray technology. *Fertil Steril* **80:** (2) 282.
Catherino WH, Segars JH. 2003. Microarray analysis in fibroids: Which gene list is the correct list? *Fertil Steril* **80:** (2) 293.
Cheng RYS, Zhao AL, Alvord WG, Powell DA, Bare RM, Masuda A, Takahashi T, Anderson LM, Kasprzak KS. 2003. Gene expression dose-response changes in microarrays after exposure of human peripheral lung epithelial cells to nickel(II). *Toxicol Appl Pharmacol* **191:** (1) 22.
Daigo Y, Takayama I, Ponder BAJ, Caldas C, Ward SM, Sanders KM, Fujino MA. 2003. Differential gene expression profile in the small intestines of mice lacking pacemaker interstitial cells of Cajal. *BMC Gastroenterol* **3:** (1) 17.
Donauer J, Rumberger B, Klein M, Faller D, Wilpert J, Sparna T, Schieren G, Rohrbach R, Dern P, Timmer J *et al*. 2003. Expression profiling on chronically rejected transplant kidneys. *Transplantation* **76:** (3) 539.
Dwek MV, Alaiya AA. 2003. Proteome analysis enables separate clustering of normal breast, benign breast and breast cancer tissues. *Br J Cancer* **89:** (2) 305.
Dyck PA, Hoda F, Osmer ES, Green RM. 2003. Microarray analysis of hepatic gene expression in gallstone-susceptible and gallstone-resistant mice. *Mamm Genome* **14:** (9) 601.
Efferth T, Gebhart E, Ross DD, Sauerbrey A. 2003. Identification of gene expression profiles predicting tumor cell response to L-alanosine. *Biochem Pharmacol* **66:** (4) 613.
Erickson LM, Pan F, Ebbs A, Kobayashi M, Jiang HS. 2003. Microarray-based gene expression profiles of allograft rejection and immunosuppression in the rat heart transplantation model. *Transplantation* **76:** (3) 582.
Franzen B, Duvefelt K, Jonsson C, Engelhardt B, Ottervald J, Wickman M, Yang Y, Schuppe-Koistinen I. 2003. Gene and protein expression profiling of human cerebral endothelial cells activated with tumor necrosis factor-α. *Mol Brain Res* **115:** (2) 130.
Freidin MB, Kobyakova O, Ogorodova LM, Puzyrev VP. 2003. Association of polymorphisms in the human IL4 and IL5 genes with atopic bronchial asthma and severity of the disease. *Comp Funct Genom* **4:** (3) 346.
Freije WA. 2003. Genome biology and gynecology: The application of oligonucleotide microarrays to leiomyomata. *Fertil Steril* **80:** (2) 277.
Gunther EC, Stone DJ, Gerwien RW, Bento P, Heyes MP. 2003. Prediction of clinical drug efficacy by classification of drug-induced genomic expression profiles *in vitro*. *Proc Natl Acad Sci U S A* **100:** (16) 9608.
Higgins MA, Berridge BR, Mills BJ, Schultze AE, Gao H, Searfoss GH, Baker TK, Ryan TP. 2003. Gene expression analysis of the acute phase response using a canine microarray. *Toxicol Sci* **74:** (2) 470.
Kaneta Y, Kagami Y, Tsunoda T, Ohno R, Nakamura Y, Katagiri T. 2003. Genome-wide analysis of gene-expression profiles in chronic myeloid leukemia cells using a cDNA microarray. *Int J Oncol* **23:** (3) 681.
Kees UR, Ford J, Watson M, Murch A, Ringneri M, Walker RL, Meltzer P. 2003. Gene expression profiles in a panel of childhood leukemia cell lines mirror critical features of the disease. *Mol Cancer Ther* **2:** (7) 671.
Marshall J, Kupchak P, Zhu WM, Yantha J, Vrees T, Furesz S, Jacks K, Smith C, Kireeva I, Zhang R, Takahashi M, Stanton E, Jackowski G. 2003. Processing of serum proteins underlies the mass spectral fingerprinting of myocardial infarction. *J Proteome Res* **2:** (4) 361.
Ohira M, Morohashi A, Inuzuka H, Shishikura T, Kawamoto T, Kageyama H, Nakamura Y, Isogai E, Takayasu H, Sakiyama S *et al*. 2003. Expression profiling and characterization of 4200 genes cloned from primary neuroblastomas: Identification of 305 genes differentially expressed between favorable and unfavorable subsets. *Oncogene* **22:** (35) 5525.
Polan ML, Warrington JA, Chen B, Mahadevappa M, Wang HB, Wen Y. 2003. Bench to bedside: Clinical opportunities for microarray analysis. *Fertil Steril* **80:** (2) 291.
Powell CA, Spira A, Derti A, DeLisi C, Liu G, Borczuk A, Busch S, Sahasrabudhe S, Chen YD, Sugarbaker D *et al*. 2003. Gene expression

Copyright © 2004 John Wiley & Sons, Ltd. 

in lung adenocarcinomas of smokers and nonsmokers. *Am J Respir Cell Mol Biol* **29:** (2) 157.
Pusztai L, Ayers M, Stec J, Clark E, Hess K, Stivers D, Damokosh A, Sneige N, Buchholz TA, Esteva FJ *et al*. 2003. Gene expression profiles obtained from fine-needle aspirations of breast cancer reliably identify routine prognostic markers and reveal large-scale molecular differences between estrogen-negative and estrogen-posItive tumors. *Clin Cancer Res* **9:** (7) 2406.
Rainczuk A, Scorza T, Smooker PM, Spithill TW. 2003. Induction of specific T-cell responses, opsonizing antibodies, and protection against *Plasmodium chabaudi adami* infection in mice vaccinated with genomic expression libraries expressed in targeted and secretory DNA vectors. *Infect Immun* **71:** (8) 4506.
Razzouk S, Shapiro IM. 2003. Detection of apoptotic gene expression in human osteoblast-like cells by cDNA microarrays. *J Bone Miner Metab* **21:** (5) 261.
Schirmer EC, Florens L, Guan TL, Yates JR, Gerace L. 2003. Nuclear membrane proteins with potential disease links found by subtractive proteomics. *Science* **301:** (5638) 1380.
Sha N, Vannucci M, Brown PJ, Trower MK, Amphlett G, Falciani F. 2003. Gene selection in arthritis classification with large-scale microarray expression profiles. *Comp Funct Genom* **4:** (2) 171.
Singhal S, Wiewrodt R, Maiden LD, Amin KM, Matzie K, Friedberg J, Kucharczuk JC, Litzky LA, Johnson SW, Kaiser LR *et al*. 2003. Gene expression profiling of malignant mesothelioma. *Clin Cancer Res* **9:** (8) 3080.
Sinha P, Poland J, Kohl S, Schnolzer M, Helmbach H, Hutter G, Lage H, Schadendorf D. 2003. Study of the development of chemoresistance in melanoma cell lines using proteome analysis. *Electrophoresis* **24:** (14) 2386.
Tsibris JCM, Segars J, Enkemann S, Coppola D, Wilbanks GD, O'Brien WF, Spellacy WN. 2003. New and old regulators of uterine leiomyoma growth from screening with DNA arrays. *Fertil Steril* **80:** (2) 279.
Ueno S, Ohki R, Hashimoto T, Takizawa T, Takeuchi K, Yamashita Y, Ota J, Choi YL, Wada T, Koinuma K *et al*. 2003. DNA microarray analysis of *in vivo* progression mechanism of heart failure. *Biochem Biophys Res Commun* **307:** (4) 771.
Van den Boom J, Wolter M, Kuick R, Misek DE, Youkilis AS, Wechsler DS, Sommer C, Reifenberger G, Hanash SM. 2003. Characterization of gene expression profiles associated with glioma progression using oligonucleotide-based microarray analysis and real-time reverse transcription-polymerase chain reaction. *Am J Pathol* **163:** (3) 1033.
Voshol H, Glucksman MJ, Van Oostrum J. 2003. Proteomics in the discovery of new therapeutic targets for psychiatric disease. *Curr Mol Med* **3:** (5) 447.
Wang HB, Mahadevappa M, Yamamoto K, Wen Y, Chen B, Warrington JA, Polan ML. 2003. Distinctive proliferative phase differences in gene expression in human myometrium and leiomyomata. *Fertil Steril* **80:** (2) 266.
Wang HY, Liu SX, Lu XH, Zhao XA, Chen SY, Li XM. 2003. Gene expression profile changes in NB4 cells induced by realgar. *Chin Med J (Engl)* **16:** (7) 1074.
Wang JF, Taylor CR. 2003. Apoptosis in cell cycle-related genes and proteins in classical Hodgkin lymphoma: Application of tissue microarray technique. *Appl Immunohistochem* **11:** (3) 206.
Weichhart T, Horky M, Sollner J, Gangl S, Henics T, Nagy E, Meinke A, Von Gabain A, Fraser CM, Gill SR *et al*. 2003. Functional selection of vaccine candidate peptides from *Staphylococcus aureus* whole-genome expression libraries *in vitro*. *Infect Immun* **71:** (8) 4633.
Weston G, Trajstman AC, Gargett CE, Manuelpillai U, Vollenhoven BJ, Rogers PAW. 2003. Fibroids display an anti-angiogenic gene expression profile when compared with adjacent myometrium. *Mol Hum Reprod* **9:** (9) 541.
Wilson KHS, Eckenrode SE, Li QZ, Ruan QG, Yang P, Shi JD, Davoodi-Semiromi A, McIndoe RA, Croker BP, She JX. 2003. Microarray analysis of gene expression in the kidneys of new- and post-onset diabetic NOD mice. *Diabetes* **52:** (8) 2151.
Xie JP, Li Y, Yue J, Xu YZ, Huang DQ, Liang L, Wang HH. 2003. Exploring *Mycobacterium tuberculosis* infection-induced alterations in gene expression in macrophage by microarray hybridization. *Sci China C Life Sci* **46:** (4) 337.
Xu YZ, Xie JP, Li Y, Yue J, Chen JP, Chunyu LJ, Wang HH. 2003. Using a cDNA microarray to study cellular gene expression altered by *Mycobacterium tuberculosis*. *Chin Med J (Engl)* **116:** (7) 1070.
Yagi T, Morimoto A, Eguchi M, Hibi S, Sako M, Ishii E, Mizutani S, Imashuku S, Ohki M, Ichikawa H. 2003. Identification of a gene expression signature associated with pediatric AML prognosis. *Blood* **102:** (5) 1849.
Yanagisawa K, Shyr Y, Xu BGJ, Massion PP, Larsen PH, White BC, Roberts JR, Edgerton M, Gonzalez A, Nadaf S, Moore JH, Caprioli RM, Carbone DP. 2003. Proteomic patterns of tumour subsets in non-small-cell lung cancer. *Lancet* **362:** (9382) 433.
Ye B, Cramer DW, Skates SJ, Gygi SP, Pratomo V, Fu LF, Horick NK, Licklider LJ, Schorge JO, Berkowitz RS, Mok SC. 2003. Haptoglobin-α subunit as potential serum biomarker in ovarian cancer: Identification and characterization using proteomic profiling and mass spectrometry. *Clin Cancer Res* **9:** (8) 2904.
Zeschnigk M, Tschentscher F, Lich C, Brandt B, Horsthemke B, Lohmann DR. 2003. Methylation analysis of several tumour suppressor genes shows a low frequency of methylation of CDKN2A and RARB in uveal melanomas. *Comp Funct Genom* **4:** (3) 329.
Zhang BC, Nie XM, Xiao BY, Xiang JJ, Shen SR, Gong JL, Zhou M, Zhu SG, Zhou J, Qian J, et al. 2003. Identification of tissue-specific genes in nasopharyngeal epithelial tissue and differentially expressed genes in nasopharyngeal carcinoma by suppression subtractive hybridization and cDNA microarray. *Gene Chromosomes Cancer* **38:** (1) 80.

### 8 EST, cDNA and other clone resources

Katoh A, Yamaguchi Y, Sano H, Hashimoto T. 2003. Analysis of expression sequence tags from *Nicotiana sylvestris*. *Proc Jpn Acad B* **79:** (6) 151.
Komulainen P, Brown GR, Mikkonen M, Karhu A, Garcia-Gil MR, O'Malley D, Lee B, Neale DB, Savolainen O. 2003. Comparing EST-based genetic maps between *Pinus sylvestris* and *Pinus taeda*. *Theor Appl Genet* **107:** (4) 667.
Scheetz TE, Trivedi N, Roberts CA, Kucaba T, Berger B, Robinson NL, Birkett CL, Gavin AJ, O'Leary B, Braun TA *et al*. 2003. ESTprep: Preprocessing cDNA sequence reads. *Bioinformatics* **19:** (11) 1318.
Yang TJ, Yu Y, Nah G, Atkins M, Lee S, Frisch DA, Wing RA. 2003. Construction and utility of 10-kb libraries for efficient clone-gap closure for rice genome sequencing. *Theor Appl Genet* **107:** (4) 652.

### 9 Functional genomics

Bjarnason J, Southward CM, Surette MG. 2003. Genomic profiling of iron-responsive genes in *Salmonella enterica* serovar Typhimurium by high-throughput screening of a random promoter library. *J Bacteriol* **185:** (16) 4973.
Griffith JL, Coleman LE, Raymond AS, Goodson SG, Pittard WS, Tsui C, Devine SE. 2003. Functional genomics reveals relationships between the retrovirus-like Ty1 element and its host *Saccharomyces cerevisiae*. *Genetics* **164:** (3) 867.
Guccione S, Yang YS, Shi GY, Lee DY, Li KCP, Bednarski MD. 2003. Functional genomics guided with MR imaging: Mouse tumor model study. *Radiology* **228:** (2) 560.
Hansen J, Floss T, Van Sloun P, Fuchtbauer EM, Vauti F, Arnold HH, Schnutgen F, Wurst W, Von Melchner H, Ruiz P. 2003. A large-scale, gene-driven mutagenesis approach for the functional analysis of the mouse genome. *Proc Natl Acad Sci U S A* **100:** (17) 9918.
Iliopoulos I, Enright AJ, Poullet P, Ouzounis CA. 2003. Mapping functional associations in the entire genome of *Drosophila melanogaster* using fusion analysis. *Comp Funct Genom* **4:** (3) 337.
Kile BT, Hentges KE, Clark AT, Nakamura H, Salinger AP, Liu B, Box N, Stockton DW, Johnson RL, Behringer RR *et al*. 2003. Functional genetic analysis of mouse chromosome 11. *Nature* **425:** (6953) 81.
Vastenhouw NL, Fischer SEJ, Robert VJP, Thijssen KL, Fraser AG, Kamath RS, Ahringer J, Plasterk RHA. 2003. A genome-wide screen identifies 27 genes involved in transposon silencing in *C. elegans*. *Curr Biol* **13:** (15) 1311.
Wu CY, Li XJ, Yuan WY, Chen GX, Kilian A, Li J, Xu CG, Li XH, Zhou DX, Wang SP, Zhang OF. 2003. Development of enhancer trap lines for functional analysis of the rice genome. *Plant J* **35:** (3) 418.
Yamamoto YY, Tsuhara Y, Gohda K, Suzuki K, Matsui M. 2003. Gene trapping of the *Arabidopsis* genome with a firefly luciferase reporter. *Plant J* **35:** (2) 273.

Copyright © 2004 John Wiley & Sons, Ltd. 

### 10 Transcriptomics

Alon T, Friedman JM, Socci ND. 2003. Cytokine-induced patterns of gene expression in skeletal muscle tissue. *J Biol Chem* **278:** (34) 32324.

Bittel DC, Kibiryeva N, Talebizadeh Z, Butler MG. 2003. Microarray analysis of gene/transcript expression in Prader-Willi syndrome: Deletion *versus* UPD. *J Med Genet* **40:** (8) 568.

Casati P, Walbot V. 2003. Gene expression profiling in response to ultraviolet radiation in maize genotypes with varying flavonoid content. *Plant Physiol* **132:** (4) 1739.

Choi JH, Lee SJ, Lee SJ, Lee SY. 2003. Enhanced production of insulin-like growth factor I fusion protein in *Escherichia coli* by coexpression of the down-regulated genes identified by transcriptome profiling. *Appl Environ Microbiol* **69:** (8) 4737.

Connolly SB, Sadlier D, Kieran NE, Doran P, Brady HR. 2003. Transcriptome profiling and the pathogenesis of diabetic complications. *J Am Soc Nephrol* **14:** (Suppl 3) S279.

Damon M, Liaubet L, Vincent A, Herpin P, Hatey F. 2003. Development of skeletal muscle transcriptome analysis to study pig meat quality. *Sci Aliment* **23:** (1) 79.

Fukumura R, Takahashi H, Saito T, Tsutsumi Y, Fujimori A, Sato S, Tatsumi K, Araki R, Abe M. 2003. A sensitive transcriptome analysis method that can detect unknown transcripts: Online art. no. e94. *Nucleic Acids Res* **31:** (16) e94.

Goetsch SC, Hawke TJ, Gallardo TD, Richardson JA, Garry DJ. 2003. Transcriptional profiling and regulation of the extracellular matrix during muscle regeneration. *Physiol Genomics* **14:** (3) 261.

Grifantini R, Sebastian S, Frigimelica E, Draghi M, Bartolini E, Muzzi A, Rappuoli R, Grandi G, Genco CA. 2003. Identification of iron-activated and -repressed Fur-dependent genes by transcriptome analysis of *Neisseria meningitidis* group B. *Proc Natl Acad Sci U S A* **100:** (16) 9542.

Hot D, Antoine R, Renauld-Mongenie G, Caro V, Hennuy B, Levillain E, Huot L, Wittmann G, Poncet D, Jacob-Dubuisson F *et al*. 2003. Differential modulation of *Bordetella pertussis* virulence genes as evidenced by DNA microarray analysis. *Mol Genet Genomics* **269:** (4) 475.

Iwahashi H, Shimizu H, Odani M, Komatsu Y. 2003. Piezophysiology of genome wide gene expression levels in the yeast *Saccharomyces cerevisiae*. *Extremophiles* **7:** (4) 291.

Korn K, Gardellin P, Liao B, Amacker M, Bergstrom A, Bjorkman H, Camacho S, Dorhofer S, Dorre K, Enstrom J *et al*. 2003. Gene expression analysis using single molecule detection: Online art. no. e89. *Nucleic Acids Res* **31:** (16) e89.

Morgan KT, Casey W, Easton M, Creech D, Ni H, Yoon L, Anderson S, Qualls CW, Crosby LM, MacPherson A *et al*. 2003. Frequent sampling reveals dynamic responses by the transcriptome to routine media replacement in HepG2 cells. *Toxicol Pathol* **31:** (4) 448.

Nakamura T, Furuhashi Y, Hasegawa K, Hashimoto H, Watanabe K, Obokata J, Sugita M, Sugiura M. 2003. Array-based analysis on tobacco plastid transcripts: Preparation of a genomic microarray containing all genes and all intergenic regions. *Plant Cell Physiol* **44:** (8) 861.

Nogueira FTS, De Rosa VE, Menossi M, Ulian EC, Arruda P. 2003. RNA expression profiles and data mining of sugarcane response to low temperature. *Plant Physiol* **132:** (4) 1811.

Pedra JHF, Brandt A, Westerman R, Lobo N, Li HM, Romero-Severson J, Murdock LL, Pittendrigh BR. 2003. Transcriptome analysis of the cowpea weevil bruchid: identification of putative proteinases and α-amylases associated with food breakdown. *Insect Mol Biol* **12:** (4) 405.

Pichler FB, Laurenson S, Williams LC, Dodd A, Copp BR, Love DR. 2003. Chemical discovery and global gene expression analysis in zebrafish. *Nat Biotechnol* **21:** (8) 879.

Schworer CM, Masker KK, Wood GC, Carey DJ. 2003. Microarray analysis of gene expression in proliferating Schwann cells: Synergistic response of a specific subset of genes to the mitogenic action of heregulin plus forskolin. *J Neurosci Res* **73:** (4) 456.

Seidel SD, Kan HL, Stott WT, Schisler MR, Gollapudi BB. 2003. Identification of transcriptome profiles for the DNA-damaging agents bleomycin and hydrogen peroxide in L5178Y mouse lymphoma cells. *Environ Mol Mutagen* **42:** (1) 19.

Singh AK, McIntyre LM, Sherman LA. 2003. Microarray analysis of the genome-wide response to iron deficiency and iron reconstitution in the cyanobacterium *Synechocystis* sp PCC 6803. *Plant Physiol* **132:** (4) 1825.

Sudre K, Cassar-Malek I, Leroux C, Listrat A, Ueda Y, Jurie C, Renand G, Martin P, Hocquette JF. 2003. Transcriptome analysis of muscle in order to identify genes which determine muscle characteristics and sensory quality traits of beef. *Sci Aliment* **23:** (1) 65.

Tan YF, Li FX, Piao YS, Sun XY, Wang YL. 2003. Global gene profiling analysis of mouse uterus during the oestrous cycle. *Reproduction* **126:** (2) 171.

Valenzuela JG, Francischetti IMB, Pham VM, Garfield MK, Ribeiro JMC. 2003. Exploring the salivary gland transcriptome and proteome of the *Anopheles stephensi* mosquito. *Insect Biochem Mol Biol* **33:** (7) 717.

Weyrich AS, Zimmerman GA. 2003. Evaluating the relevance of the platelet transcriptome. *Blood* **102:** (4) 1550.

Yu HY, Luscombe NM, Qian J, Gerstein M. 2003. Genomic analysis of gene expression relationships in transcriptional regulatory networks. *Trends Genet* **19:** (8) 422.

### 11 Proteomics

Bandara LR, Kelly MD, Lock EA, Kennedy S. 2003. A potential biomarker of kidney damage identified by proteomics: Preliminary findings. *Biomarkers* **8:** (3-4) 272.

Buscemi N, Doherty-Kirby A, Sussman MA, Lajoie G, Van Eyk JE. 2003. Proteomic analysis of Rac1 transgenic mice displaying dilated cardiomyopathy reveals an increase in creative kinase M-chain protein abundance. *Mol Cell Biochem* **251:** (1-2) 145.

Chalmers MJ, Quinn JP, Blakney GT, Emmett MR, Mischak H, Gaskell SJ, Marshall AG. 2003. Liquid chromatography-Fourier transform ion cyclotron resonance mass spectrometric characterization of protein kinase C phosphorylation. *J Proteome Res* **2:** (4) 373.

Chen HC, Teplitski M, Robinson JB, Rolfe BG, Bauer WD. 2003. Proteomic analysis of wild-type *Sinorhizobium meliloti* responses to *N*-acyl homoserine lactone quorum-sensing signals and the transition to stationary phase. *J Bacteriol* **185:** (17) 5029.

Corbin RW, Paliy O, Yang F, Shabanowitz J, Platt M, Lyons CE, Root K, McAuliffe J, Jordan MI, Kustu S *et al*. 2003. Toward a protein profile of *Escherichia coli*: Comparison to its transcription profile. *Proc Natl Acad Sci U S A* **100:** (16) 9232.

Doolan DL, Southwood S, Freilich DA, Sidney J, Graber NL, Shatney L, Bebris L, Florens L, Dobano C, Witney AA *et al*. 2003. Identification of *Plasmodium falciparum* antigens by antigenic analysis of genomic and proteomic data. *Proc Natl Acad Sci U S A* **100:** (17) 9952.

Finnie C, Svensson B. 2003. Feasibility study of a tissue-specific approach to barley proteome analysis: Aleurone layer, endosperm, embryo and single seeds. *J Cereal Sci* **38:** (2) 217.

Froehlich JE, Wilkerson CG, Ray WK, McAndrew RS, Osteryoung KW, Gage DA, Phinney BS. 2003. Proteomic study of the *Arabidopsis thaliana* chloroplastic envelope membrane utilizing alternatives to traditional two-dimensional electrophoresis. *J Proteome Res* **2:** (4) 413.

Gianazza E, Veber D, Eberini I, Buccellato FR, Mutti E, Sironi L, Scalabrino G. 2003. Cobalamin (vitamin $B_{12}$)-deficiency-induced changes in the proteome of rat cerebrospinal fluid. *Biochem J* **374:** (1) 239.

Gururaja T, Li WQ, Noble WS, Payan DG, Anderson DC. 2003. Multiple functional categories of proteins identified in an *in vitro* cellular ubiquitin affinity extract using shotgun peptide sequencing. *J Proteome Res* **2:** (4) 394.

Haslam RP, Downie AL, Raveton M, Gallardo K, Job D, Pallett KE, John P, Parry MAJ, Coleman JOD. 2003. The assessment of enriched apoplastic extracts using proteomic approaches. *Ann Appl Biol* **143:** (1) 81.

Hebraud M. 2003. The proteomic approach for better understanding the physiology of bacteria adaptation to food plant environments. *Sci Aliment* **23:** (1) 97.

Orru S, Caputo I, D'Amato A, Ruoppolo M, Esposito C. 2003. Proteomics identification of acyl-acceptor and acyl-donor substrates for transglutaminase in a human intestinal epithelial cell line. *J Biol Chem* **278:** (34) 31766.

Peng JM, Schwartz D, Elias JE, Thoreen CC, Cheng DM, Marsischky G, Roelofs J, Finley D, Gygi SP. 2003. A proteomics approach to understanding protein ubiquitination. *Nat Biotechnol* **21:** (8) 921.

Copyright © 2004 John Wiley & Sons, Ltd. 

Sayd T, Laville E, Sante V, Monin G. 2003. Proteomic analysis of destructured pork muscle. *Sci Aliment* **23:** (1) 89.

### I2 Protein structural genomics

Rasmussen M, Jacobsson M, Bjorck L. 2003. Genome-based identification and analysis of collagen-related structural motifs in bacterial and viral proteins. *J Biol Chem* **278:** (34) 32313.

### I3 Metabolomics

Bro C, Regenberg B, Lagniel G, Labarre J, Montero-Lomeli M, Nielsen J. 2003. Transcriptional proteomic, and metabolic responses to lithium in galactose-grown yeast cells. *J Biol Chem* **278:** (34) 32141.

Somerville GA, Sayd-Salim B, Wickman JM, Raffel SJ, Kreiswirth BN, Musser JM. 2003. Correlation of acetate catabolism and growth yield in *Staphylococcus aureus*: Implications for host-pathogen interactions. *Infect Immun* **71:** (8) 4724.

Wang W, Sun JB, Hartlep M, Deckwer WD, Zeng AP. 2003. Combined use of proteomic analysis and enzyme activity assays for metabolic pathway analysis of glycerol fermentation by *Klebsiella pneumoniae*. *Biotechnol Bioeng* **83:** (5) 525.

### I4 Genomic approaches to development

Alge CS, Suppmann S, Priglinger SG, Neubauer AS, May CA, Hauck S, Welge-Lussen U, Ueffing M, Kampik A. 2003. Comparative proteome analysis of native differentiated and cultured dedifferentiated human RPE cells. *Invest Ophthalmol Vis Sci* **44:** (8) 3629.

Bouley J, Chambon C, Picard B. 2003. Proteome analysis applied to the study of muscle development and sensorial qualities of bovine meat. *Sci Aliment* **23:** (1) 75.

Clancy BM, Johnson JD, Lambert AJ, Rezvankhah S, Wong A, Resmini C, Feldman JL, Leppanen S, Pittman DD. 2003. A gene expression profile for endochondral bone formation: Oligonucleotide microarrays establish novel connections between known genes and BMP-2 induced bone formation in mouse quadriceps. *Bone* **33:** (1) 46.

Imabayashi H, Mori T, Gojo S, Kiyono T, Sugiyama T, Irie R, Isogai T, Hata J, Toyama Y, Umezawa A. 2003. Redifferentiation of dedifferentiated chondrocytes and chondrogenesis of human bone marrow stromal cells *via* chondrosphere formation with expression profiling by large-scale cDNA analysis. *Exp Cell Res* **288:** (1) 35.

Iranfar N, Fuller D, Loomis WF. 2003. Genome-wide expression analyses of gene regulation during early development of *Dictyostelium discoideum*. *Eukaryot Cell* **2:** (4) 664.

Kerim T, Imin N, Weinman JJ, Rolfe BG. 2003. Proteomic analysis reveals developmentally expressed rice homologues of grass group II pollen allergens. *Funct Plant Biol* **30:** (8) 843.

Srisuma S, Biswal SS, Mitzner WA, Gallagher SJ, Mai KH, Wagner EM. 2003. Identification of genes promoting angiogenesis in mouse lung by transcriptional profiling. *Am J Respir Cell Mol Biol* **29:** (2) 172.

### I5 Technological advances

Brock A, Horn DM, Peters EC, Shaw CM, Ericson C, Phung QT, Salomon AR. 2003. An automated matrix-assisted laser desorption/ionization quadrupole Fourier transform ion cyclotron resonance mass spectrometer for “bottom-up” proteomics. *Anal Chem* **75:** (14) 3419.

Joo WA, Lee DY, Kim CW. 2003. Development of an effective sample preparation method for the proteome analysis of body fluids using 2-D gel electrophoresis. *Biosci Biotechnol Biochem* **67:** (7) 1574.

Krijgsveld J, Ketting RF, Mahmoudi T, Johansen J, Artal-Sanz M, Verrijzer CP, Plasterk RHA, Heck AJR. 2003. Metabolic labeling of *C. elegans* and *D. melanogaster* for quantitative proteomics. *Nat Biotechnol* **21:** (8) 927.

McLean JA, Russell DH. 2003. Sub-femtomole peptide detection in ion mobility-time-of-flight mass spectrometry measurements. *J Proteome Res* **2:** (4) 427.

Ouyang Z, Takats Z, Blake TA, Gologan B, Guymon AJ, Wiseman JM, Oliver JC, Davisson VJ, Cooks RG. 2003. Preparing protein microarrays by soft-landing of mass-selected ions. *Science* **301:** (5638) 1351.

Sadygov RG, Yates JR. 2003. A hypergeometric probability model for protein identification and validation using tandem mass spectral data and protein sequence databases. *Anal Chem* **75:** (15) 3792.

Shang TQ, Ginter JM, Johnston MV, Larsen BS, McEwen CN. 2003. Carrier ampholyte-free solution isoelectric focusing as a prefractionation method for the proteomic analysis of complex protein mixtures. *Electrophoresis* **24:** (14) 2359.

Shen YF, Moore RJ, Zhao R, Blonder J, Auberry DL, Masselon C, Pasa-Tolic L, Hixson KK, Auberry KJ, Smith RD. 2003. High-efficiency on-line solid-phase extraction coupling to 15-150-μm-id column liquid chromatography for proteomic analysis. *Anal Chem* **75:** (14) 3596.

Shu G, Zeng B, Chen YP, Smith OH. 2003. Performance assessment of kernel density clustering for gene expression profile data. *Comp Funct Genom* **4:** (3) 287.

Tabuchi M, Kuramitsu Y, Nakamura K, Baba Y. 2003. Rapid subpicogram protein detection on a microchip without denaturing. *J Proteome Res* **2:** (4) 431.

Taylor S, Smith S, Windle B, Guiseppi-Elie A. 2003. Impact of surface chemistry and blocking strategies on DNA microarrays - Online art. no. e87. *Nucleic Acids Res* **31:** (16) e87.

Toriumi C, Imai K. 2003. An identification method for altered proteins in tissues utilizing fluorescence derivatization, liquid chromatography, tandem mass spectrometry, and a database-searching algorithm. *Anal Chem* **75:** (15) 3725.

Tyagarajan K, Pretzer E, Wiktorowicz JE. 2003. Thiol-reactive dyes for fluorescence labeling of proteomic samples. *Electrophoresis* **24:** (14) 2348.

Wang W, Scali M, Vignani R, Spadafora A, Sensi E, Mazzuca S, Cresti M. 2003. Protein extraction for two-dimensional electrophoresis from olive leaf, a plant tissue containing high levels of interfering compounds. *Electrophoresis* **24:** (14) 2369.

Wu SL, Choudhary G, Ramstrom M, Bergquist J, Hancock WS. 2003. Evaluation of shotgun sequencing for proteomic analysis of human plasma using HPLC coupled with either ion trap or Fourier transform mass spectrometry. *J Proteome Res* **2:** (4) 383.

Zabarovsky ER, Petrenko L, Protopopov A, Vorontsova O, Kutsenko AS, Zhao YY, Kilosanidze G, Zabarovska V, Rakhmanaliev E, Pettersson B *et al*. 2003. Restriction site tagged (RST) microarrays: A novel technique to study the species composition of complex microbial systems - Online art. no. e95. *Nucleic Acids Res* **31:** (16) e95.

Zhao YX, Zhang W, White MA, Zhao YM. 2003. Capillary high-performance liquid chromatography/mass spectrometric analysis of proteins from affinity-purified plasma membrane. *Anal Chem* **75:** (15) 3751.

### I6 Bioinformatics

Baggerly KA, Deng L, Morris JS, Aldaz CM. 2003. Differential expression in SAGE: Accounting for normal between-library variation. *Bioinformatics* **19:** (12) 1477.

Bajic VB, Seah SH. 2003. Dragon Gene Start Finder: An advanced system for finding approximate locations of the start of gene transcriptional units. *Genome Res* **13:** (8) 1923.

Banks MA, Eichert W, Olsen JB. 2003. Which genetic loci have greater population assignment power? *Bioinformatics* **19:** (11) 1436.

Bar-Joseph Z, Gerber GK, Gifford DK, Jaakkola TS, Simon I. 2003. Continuous representations of time-series gene expression data. *J Comput Biol* **10:** (3-4) 341.

Ben-Dor A, Chor B, Karp R, Yakhini Z. 2003. Discovering local structure in gene expression data: The order-preserving submatrix problem. *J Comput Biology* **10:** (3-4) 373.

Chang PC, Peck K. 2003. Design and assessment of a fast algorithm for identifying specific probes for human and mouse genes. *Bioinformatics* **19:** (11) 1311.

Cherepinsky V, Feng JW, Rajali M, Mishra B. 2003. Shrinkage-based similarity metric for cluster analysis of microarray data. *Proc Natl Acad Sci U S A* **100:** (17) 9668.

Chiang JH, Yu HC. 2003. MeKE: Discovering the functions of gene products from biomedical literature *via* sentence alignment. *Bioinformatics* **19:** (11) 1417.

De Santis TZ, Dubosarskiy I, Murray SR, Andersen GL. 2003. Compre-

Copyright © 2004 John Wiley & Sons, Ltd. 

hensive aligned sequence construction for automated design of effective probes (CASCADE-P) using 16S rDNA. *Bioinformatics* **19:** (12) 1461.

Draghici S, Kulaeva O, Hoff B, Petrov A, Shams S, Tainsky MA. 2003. Noise sampling method: An ANOVA approach allowing robust selection of differentially regulated genes measured by DNA microarrays. *Bioinformatics* **19:** (11) 1348.

Dror RO, Murnick JG, Rinaldi NJ, Marinescu VD, Rifkin RM, Young RA. 2003. Bayesian estimation of transcript levels using a general model of array measurement noise. *J Comput Biol* **10:** (3-4) 433.

Durand D, Sankoff D. 2003. Tests for gene clustering. *J Comput Biology* **10:** (3-4) 453.

Durbin B, Rocke DM. 2003. Estimation of transformation parameters for microarray data. *Bioinformatics* **19:** (11) 1360.

Edgar RC, Sjolander K. 2003. SATCHMO: Sequence alignment and tree construction using hidden Markov models. *Bioinformatics* **19:** (11) 1404.

Fang YX, Brass A, Hoyle DC, Hayes A, Bashein A, Oliver SG, Waddington D, Rattray M. 2003. A model-based analysis of microarray experimental error and normalisation: Online art. no. e96. *Nucleic Acids Res* **31:** (16) e96.

Fink JL, Drewes S, Patel H, Welsh JB, Masys DR, Corbeil J, Gribskov MR. 2003. 2HAPI: A microarray data analysis system. *Bioinformatics* **19:** (11) 1443.

Fischer TB, Arunachalam KV, Bailey D, Mangual V, Bakhru S, Russo R, Huang D, Paczkowski M, Lalchandani V, Ramachandra C *et al*. 2003. The binding interface database (BID): A compilation of amino acid hot spots in protein interfaces. *Bioinformatics* **19:** (11) 1453.

Gibson R, Smith DR. 2003. Genome visualization made fast and simple. *Bioinformatics* **19:** (11) 1449.

Grasso C, Quist M, Ke K, Lee C. 2003. POAVIZ: A partial order multiple sequence alignment visualizer. *Bioinformatics* **19:** (11) 1446.

Hoon S, Ratnapu KK, Chia J, Kumarasamy B, Xiao JG, Clamp M, Stabenau A, Potter S, Clarke L, Stupka E. 2003. Biopipe: A flexible framework for protocol-based bioinformatics analysis. *Genome Res* **13:** (8) 1904.

Hu YH, Hines LM, Weng HF, Zuo DM, Rivera M, Richardson A, LaBaer J. 2003. Analysis of genomic and proteomic data using advanced literature mining. *J Proteome Res* **2:** (4) 405.

Janssen P, Enright AJ, Audit B, Cases I, Goldovsky L, Harte N, Kunin V, Ouzounis CA. 2003. COmplete GENome Tracking (COGENT): A flexible data environment for computational genomics. *Bioinformatics* **19:** (11) 1451.

Korf I. 2003. Serial BLAST searching. *Bioinformatics* **19:** (12) 1492.

Kotlar D, Lavner Y. 2003. Gene prediction by spectral rotation measure: A new method for identifying protein-coding regions. *Genome Res* **13:** (8) 1930.

Kreil DP, MacKay DC. 2003. Reproducibility assessment of independent component analysis of expression ratios from DNA microarrays. *Comp Funct Genom* **4:** (3) 300.

Krishnamoorthy B, Tropsha A. 2003. Development of a four-body statistical pseudo-potential to discriminate native from non-native protein conformations. *Bioinformatics* **19:** (12) 1540.

Kunin V, Ouzounis CA. 2003. GeneTRACE-reconstruction of gene content of ancestral species. *Bioinformatics* **19:** (11) 1412.

Lambrix P, Habbouche M, Perez M. 2003. Evaluation of ontology development tools for bioinformatics. *Bioinformatics* **19:** (12) 1564.

Langmead CJ, Yan AK, McClung CR, Donald BR. 2003. Phase-independent rhythmic analysis of genome-wide expression patterns. *J Comput Biology* **10:** (3-4) 521.

Li KB. 2003. ClustalW-MPI: ClustalW analysis using distributed and parallel computing. *Bioinformatics* **19:** (12) 1585.

Li Y, Rosso MG, Strizhov N, Viehoever P, Weisshaar B. 2003. GABI-Kat SimpleSearch: A flanking sequence tag (FST) database for the identification of T-DNA insertion mutants in *Arabidopsis thaliana*. *Bioinformatics* **19:** (11) 1441.

Loytynoja A, Milinkovitch MC. 2003. A hidden Markov model for progressive multiple alignment. *Bioinformatics* **19:** (12) 1505.

Luo L, Li H, Zhang L. 2003. ORF organization and gene recognition in the yeast genome. *Comp Funct Genom* **4:** (3) 318.

Ma HW, Zeng AP. 2003. The connectivity structure, giant strong component and centrality of metabolic networks. *Bioinformatics* **19:** (11) 1423.

Madden SF, O'Donovan B, Furney SJ, Brady HR, Silvestre G, Doran PP. 2003. Digital extractor: Analysis of digital differential display output. *Bioinformatics* **19:** (12) 1594.

Marengo E, Leardi R, Robotti E, Righetti PG, Antonucci F, Cecconi D. 2003. Application of three-way principal component analysis to the evaluation of two-dimensional maps in proteomics. *J Proteome Res* **2:** (4) 351.

Mathura VS, Schein CH, Braun W. 2003. Identifying property based sequence motifs in protein families and superfamilies: Application to DNase-1 related endonucleases. *Bioinformatics* **19:** (11) 1381.

Mittelman D, Sadreyev R, Grishin N. 2003. Probabilistic scoring measures for profile-profile comparison yield more accurate short seed alignments. *Bioinformatics* **19:** (12) 1531.

Pan W. 2003. On the use of permutation in and the performance of a class of nonparametric methods to detect differential gene expression. *Bioinformatics* **19:** (11) 1333.

Paquola ACM, Nishyiama MY, Reis EM, Da Silva AM, Verjovski-Almeida S. 2003. ESTWeb: Bioinformatics services for EST sequencing projects. *Bioinformatics* **19:** (12) 1587.

Rajagopalan D. 2003. A comparison of statistical methods for analysis of high density oligonucleotide array data. *Bioinformatics* **19:** (12) 1469.

Ronquist F, Huelsenbeck JP. 2003. MrBayes 3: Bayesian phylogenetic inference under mixed models. *Bioinformatics* **19:** (12) 1572.

Sammeth M, Rothganger J, Esser W, Albert J, Stoye J, Harmsen D. 2003. QAlign: Quality-based multiple alignments with dynamic phylogenetic analysis. *Bioinformatics* **19:** (12) 1592.

Sato N, Ehira S. 2003. GenoMap, a circular genome data viewer. *Bioinformatics* **19:** (12) 1583.

Shih ACC, Li WH. 2003. GS-aligner: A novel tool for aligning genomic sequences using bit-level operations. *Mol Biol Evol* **20:** (8) 1299.

Simon P. 2003. Q-Gene: Processing quantitative real-time RT-PCR data. *Bioinformatics* **19:** (11) 1439.

Somorjai RL, Dolenko B, Baumgartner R. 2003. Class prediction and discovery using gene microarray and proteomics mass spectroscopy data: Curses, caveats, cautions. *Bioinformatics* **19:** (12) 1484.

Su Y, Murali TM, Pavlovic V, Schaffer M, Kasif S. 2003. RankGene: Identification of diagnostic genes based on expression data. *Bioinformatics* **19:** (12) 1578.

Subramanian S, Madgula VM, George R, Kumar S, Pandit MW, Singh L. 2003. SSRD: Simple sequence repeats database of the human genome. *Comp Funct Genom* **4:** (3) 342.

Taher L, Rinner O, Garg S, Sczyrba A, Brudno M, Batzoglou S, Morgenstern B. 2003. AGenDA: Homology-based gene prediction. *Bioinformatics* **19:** (12) 1575.

Valafar H, Prestegard JH. 2003. Rapid classification of a protein fold family using a statistical analysis of dipolar couplings. *Bioinformatics* **19:** (12) 1549.

Van Hijum SAFT, De Jong A, Buist G, Kok J, Kuipers OP. 2003. UniFrag and GenomePrimer: Selection of primers for genome-wide production of unique amplicons. *Bioinformatics* **19:** (12) 1580.

Wang GL, Dunbrack RL. 2003. PISCES: A protein sequence culling server. *Bioinformatics* **19:** (12) 1589.

Wang XJ, Hessner MJ, Wu Y, Pati N, Ghosh S. 2003. Quantitative quality control in microarray experiments and the application in data filtering normalization and false positive rate prediction. *Bioinformatics* **19:** (11) 1341.

Webber C, Barton GJ. 2003. Increased coverage obtained by combination of methods for protein sequence database searching. *Bioinformatics* **19:** (11) 1397.

Weinel C, Ermolaeva MD, Ouzounis C. 2003. PseuRECA: Genome annotation and gene context analysis for *Pseudomonas aeruginosa* PAO1. *Bioinformatics* **19:** (12) 1457.

Whelan S, De Bakker PIW, Goldman N. 2003. Pandit: A database of protein and associated nucleotide domains with inferred trees. *Bioinformatics* **19:** (12) 1556.

Wilson DL, Buckley MJ, Helliwell CA, Wilson IW. 2003. New normalization methods for cDNA microarray data. *Bioinformatics* **19:** (11) 1325.

Wu J, Kasif S, DeLisi C. 2003. Identification of functional links between genes using phylogenetic profiles. *Bioinformatics* **19:** (12) 1524.

Zhang HY. 2003. Alignment of BLAST high-scoring segment pairs based on the longest increasing subsequence algorithm. *Bioinformatics* **19:** (11) 1391.

Zhang LX, Ma B, Wang LS, Xu Y. 2003. Greedy method for inferring tandem duplication history. *Bioinformatics* **19:** (12) 1497.

Copyright © 2004 John Wiley & Sons, Ltd.
